# A biogeographical study on tropical flora of southern China

**DOI:** 10.1002/ece3.3561

**Published:** 2017-10-30

**Authors:** Hua Zhu

**Affiliations:** ^1^ Center for Integrative Conservation Xishuangbanna Tropical Botanical Garden Chinese Academy of Sciences Mengla Yunnan China

**Keywords:** floristic affinity, floristic composition, geographical elements, geological histories, southern China, tropical flora

## Abstract

The tropical climate in China exists in southeastern Xizang (Tibet), southwestern to southeastern Yunnan, southwestern Guangxi, southern Guangdon, southern Taiwan, and Hainan, and these southern Chinese areas contain tropical floras. I checked and synonymized native seed plants from these tropical areas in China and recognized 12,844 species of seed plants included in 2,181 genera and 227 families. In the tropical flora of southern China, the families are mainly distributed in tropical areas and extend into temperate zones and contribute to the majority of the taxa present. The genera with tropical distributions also make up the most of the total flora. In terms of geographical elements, the genera with tropical Asian distribution constitute the highest proportion, which implies tropical Asian or Indo‐Malaysia affinity. Floristic composition and geographical elements are conspicuous from region to region due to different geological history and ecological environments, although floristic similarities from these regions are more than 90% and 64% at the family and generic levels, respectively, but lower than 50% at specific level. These differences in the regional floras could be influenced by historical events associated with the uplift of the Himalayas, such as the southeastward extrusion of the Indochina geoblock, clockwise rotation and southeastward movement of Lanping–Simao geoblock, and southeastward movement of Hainan Island. The similarity coefficients between the flora of southern China and those of Indochina countries are more than 96% and 80% at family and generic levels, indicating their close floristic affinity and inclusion in the same biogeographically floristic unit.

## 1 INTRODUCTION

The existence of tropical flora and distinct tropical rain forest vegetation in the biogeography of southwestern China was first pointed out by Fedorov (1957, 1958); however, Chinese botanist Wu (1965) clarified the tropical affinity of the flora of China based on the analysis of the geographical elements associated with Chinese seed plants at the generic level. Whitmore (1982, 1990) confirmed the presence of the Southeast Asian rain forest in southern China with a short visit in 1980. Species‐rich tropical forests, which exist along much of China's southern border, were found to share conspicuous ecological and floristic similarity to forests in SE Asia (Zhu, 1997, 2008a,b, 2017a; Zhu, Cao, & Hu, 2006). The earliest compiled local flora for the tropical areas of China was made for Hainan Island (South China Botanical Institute, 1964–1977), but there is a need for a panoramic work to fully understand the biodiversity of the tropical flora of China, especially for conservation efforts. In this article, the floristic composition, geographical elements, floristic variation, and possible evolution of the tropical flora of China are investigated, and the possible influences by geohistorical events associated with the uplift of the Himalayas have been also discussed.

## 2 GEOGRAPHY

**Figure 1:**
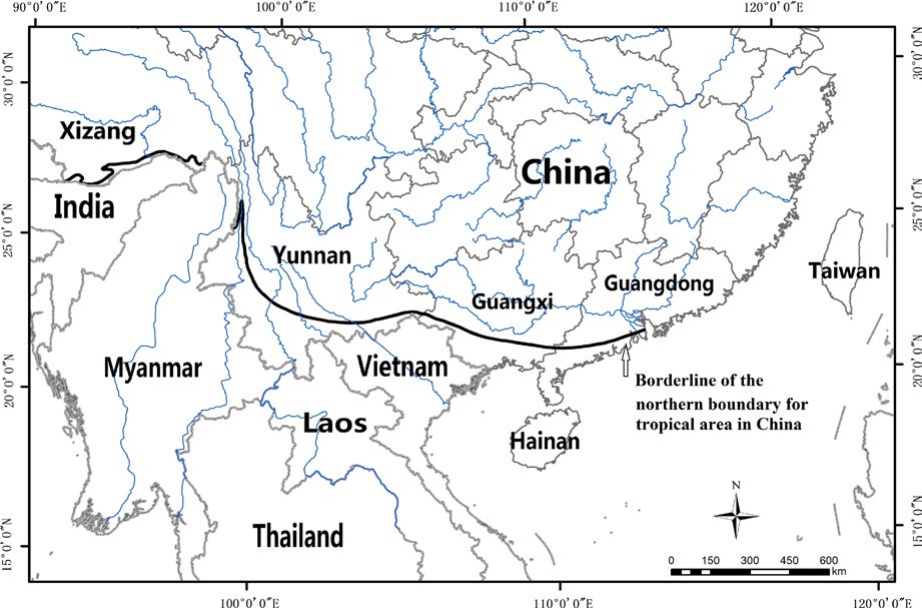
Borderline suggested as the biogeographically northern boundary for the tropical area in south and southeastern China. (The figure was made by the Landscape Ecology Lab., Xishuangbanna Tropical Botanical Garden, CAS)

Areas with a tropical climate in China include southeastern Xizang (Tibet), southwestern to southeastern Yunnan, southwestern Guangxi, southern Guangdon, southern Taiwan, and Hainan. Both climatic and physical zonation indicate that the tropical zone is generally south of the Tropic of Cancer, with the exception of parts of southwest China (National Committee of Atlas Compilations, 1999), although the precise demarcation line for the tropical area has been debatable and varyingly applied. A line at c. 22°30′N was tentatively suggested as the northern boundary of the tropical zone in south and southeastern China based on the biogeographical patterns of Chinese seed plants, such as the dominance of tropical genera in this area (Zhu, 2013a; Zhu, Ma, Yan, & Hu, 2007). This line corresponds well with the currently recognized northern boundary of the tropical monsoon and rain forests of China (Wu, 1980; Zhang, 2007; Zhu, 2017a). Despite a slightly lower annual cumulative temperature in the region of southern China surrounding c. 22°30′N, tropical genera account for more than 80% of the total genera in the lowland floras and exhibit a Southeast Asian floristic affinity (Zhu, 2008a,b, 2013a; Zhu & Roos, 2004; Zhu et al., 2007). This line also coincides with the demarcation between two established floristic regions in China, the East Asiatic Kingdom (Wu & Wu, 1996) or Holarctic Kingdom, and the Paleotropical Kingdom (Takhtajan, 1978). The location of a boundary at c. 22°30′N is additionally supported by a similar periphery line drawn by Ashton (2014) to distinguish major zonal forest formations of lowland tropical Asia based on herbarium specimens and personal experience. I suggest that using the 22°30′N borderline is a suitable biogeographical boundary for the tropical areas in south and southeastern China, see Figure 1.

## 3 MATERIALS AND METHODS

Based on the biogeographical boundary for the tropical areas in south and southeastern China, data on the respective tropical floras from these southern China provinces were collected: Motuo of Xizang (Yang & Zhou, 2015), southern Yunnan (Zhu & Yan, 2012), southeastern Yunnan (Zhu & Yan, 2009), southwestern Guangxi (Qin & Liu, 2010), and Hainan (Xing, Zhou, Wang, Zeng, & Liu, 2012). I checked and synonymized the native seed plants from these tropical areas in China, and was able to recognize 12,844 species of seed plants comprising of 2,181 genera in 227 families (not including Taiwan due to the lack of a tropical plant checklist) (see Appendix S1 and S2). The circumscriptions of families followed the APG III and APG IV technique (APG, 2009, 2016; Chase & Reveal, 2009), and I followed w^3^TROPICOS ( http://mobot.mobot.org/W3T/Search/vast.html) for species nomenclature. Patterns of seed plant distribution were quantified at the generic and the family levels following Wu (1991) and Wu, Zhou, Sun, Li, and Peng (2006) and placed in the following categories: cosmopolitan, pantropic, tropical Asia and tropical America disjuncted, Old World tropics, tropical Asia to tropical Australia, tropical Asia to tropical Africa, tropical Asia, north temperate, East Asia and North America disjuncted, Old World temperate, temperate Asia, Mediterranean region, West to Central Asia, Central Asia, East Asia, and Endemic to China. The biogeographical affinity of the flora was investigated using geographical elements at the family and generic levels. For further understanding, both the tropical Asian affinity of the Chinese tropical flora and comparisons between the tropical flora of China and Indochina peninsula countries are made using the data from the floras of Laos (Zhu, 2017b), Myanmar (Kress, DePilipps, Farr, & Kyi, 2003), Thailand (Smitinand, 2001), and Vietnam (Chan, 1999; Zhu, Yan, & Qin, 2003). As the usual, I use the similarity coefficients at family and generic levels to clarify the affinity between these regional floras in this article.

## 4 RESULTS

### 4.1 Floristic composition

I was able to recognize 12,844 species of seed plants comprising of 2,181 genera and 227 families from the tropical areas of southern China. Among them were 14 families, which comprised of more than 200 species each, such as Ochidaceae (823 species/153 genera), Fabaceae (692 species/118 genera), Poaceae (630/170), Rubiaceae (534/81), Asteraceae (484/114), Lamiaceae (398/75), Lauraceae (345/18), and Euphorbiaceae (335/62); 23 families with 100–200 species, such as Gesneriaceae (186/34), Moraceae (183/10), Asclepiadaceae (179/41), Theaceae (169/13), Zingiberaceae (167/20), Araliaceae (145/21), Vitaceae (142/9), and Rutaceae (136/20) (Table 1).

**Table 1:** Dominant (top 30) families in species richness with their distribution

Family ranking by their species richness	Number of genus	Number of species	Distribution type^a^
Orchidaceae	153	832	1
Fabaceae	118	692	2
Poaceae	170	630	1
Rubiaceae	81	534	1
Asteraceae	114	484	1
Lamiaceae	75	398	1
Lauraceae	18	345	2
Euphorbiaceae	62	335	2
Rosaceae	36	329	1
Cyperaceae	33	301	1
Urticaceae	24	274	2
Ericaceae	14	257	1
Fagaceae	8	248	8
Acanthaceae	56	221	2
Gesneriaceae	34	186	3
Moraceae	10	183	1
Asclepiadaceae	41	179	2
Theaceae	13	169	2
Zingiberaceae	20	167	5
Araliaceae	21	145	3
Vitaceae	9	142	2
Rutaceae	20	136	2
Celastraceae	12	133	2
Scrophulariaceae	35	131	1
Cucurbitaceae	26	128	2
Ranunculaceae	20	128	1
Apocynaceae	31	127	2
Araceae	28	124	2
Melastomataceae	19	124	2
Myrsinaceae	5	122	2

a Distribution type: 1: cosmopolitan, 2: pantropic, 3 tropical Asia and tropical America disjuncted, 5: tropical Asia to tropical Australia, 8: north temperate.

The majority of the families with more than 200 species were determined to have a cosmopolitan distribution, but families with 100–200 species generally showed a pantropic distribution. I found that less species‐rich families were dominant and characteristic in the tree canopy layers of the southern China tropical forests, and exhibited a strict tropical distribution. These families were as follows: Sapingdaceae, Anacardiaceae, Burseraceae, Elaeocarpaceae, Ebenaceae, Combretaceae, Myrtaceae, Clusiaceae, Dilleniaceae, Dipterocarpaceae, Myristicaceae, Icacinaceae, Ixonanthaceae, Sapotaceae, Sterculiaceae.

The species‐rich genera in Chinese tropical floras are *Ficus* (131 species), *Rhododendron* (112 species, mainly in southeastern Xizang and southeastern Yunnan), *Rubus* (102), *Elatostema* (100 species)*, Lithocarpus* (99), *Ilex* (95), *Bulbophyllum* (74), *Litsea* (70), *Dendrobium* (67), *Syzygium* (66), *Ardisia* (62), *Camellia* (62), *Piper* (62), and *Tetrastigma* (61). Similar to the geographical patterns of families, I found that the majority of species‐rich genera presented a pantropic distribution, but did extend into temperate areas.

### 4.2 Biogeographical elements

The pantropic distribution at the family level makes up the highest ratio, with 86 families representing 37.89% of the total. Further, we found that cosmopolitan families make up 20.70%, and the north temperate families make up 13.66% (Table 2). The total tropical distributions (Type 2‐7) consisted of 132 families, contributing to 58.15%, whereas the temperate distributions totaled (Type 8‐15) 48 families, contributing only 21.15%.

**Table 2:** Areal types at family level in the tropical flora of southern China

Areal types of family	No. of family	%
1 Cosmopolitan	47	20.70
2 Pantropic	86	37.89
3 Tropical Asia and tropical America disjuncted	14	6.17
4 Old World tropics	10	4.41
5 Tropical Asia to tropical Australasia	9	3.96
6 Tropical Asia to tropical Africa	3	1.32
7 Tropical Asia (Indo‐Malaysia)	10	4.41
2–7 (Total tropical elements)	(132)	(58.15)
8 North temperate	31	13.66
9 E. Asia and N. America disjuncted	9	3.96
10 Old World temperate	1	0.44
11 Temperate Asia	0	0.00
12 Mediterranean, West Asia to Central Asia	0	0.00
13 Central Asia	0	0.00
14 East Asia	7	3.08
15 Endemic to China	0	0.00
8–15 (Total temperate elements)	(48)	(21.15)
Total	227	100.00

At the generic level, tropical distributions (Type 2‐7) contribute to 67.22% (Table 3); among them, the tropical Asian distribution had the highest ratio, making up 26.04% of the total genera. These include *Aganosma*,* Alphonsea*,* Ammora, Aphanamixis, Chukrasia*,* Crypteronia*,* Gynostemma*,* Knema, Mitrephora, Pterospermum*. The second highest distributional class was the pantropic distribution, which made up 15.82%, and included *Beilschmiedia*,* Capparis*,* Cleidion*,* Cryptocarya, Dioscorea*,* Gnetum*,* Lasianthus*,* Marsdenia, Millettia*,* Morinda*. Old World tropical distributions were 7.66% and included *Fissistigma*,* Dracaena*,* Pandanus*,* Polyalthia, Stephania*,* Syzigium*,* Thunbergia, Ventilago*. For the tropical Asia to tropical Australasia distribution, they included *Argyreia*,* Dischidia*,* Dillenia*,* Hoya*,* Lagerstroemia*,* Loeseneriella*,* Murraya, Wendlandia* and made up 9.08%. Lastly, the distributions of tropical Asia to tropical Africa comprised of 6.01%, and included the following: *Anogeissus, Bombax*,* Flacourtia*,* Garcinia, Ixora*,* Mitragyna*,* Premna*,* Quisqualis*,* Toddalia*.

**Table 3:** Areal types at generic level in the tropical flora of southern China

Areal types of genera	No. of genus	%
1 Cosmopolitan	92	4.22
2 Pantropic	345	15.82
3 Tropical Asia and tropical America disjuncted	57	2.61
4 Old World tropics	167	7.66
5 Tropical Asia to tropical Australasia	198	9.08
6 Tropical Asia to tropical Africa	131	6.01
7 Tropical Asia (Indo‐Malaysia)	568	26.04
2–7 (Total tropical elements)	(1,466)	(67.22)
8 North temperate	190	8.71
9 East Asia and North America disjuncted	79	3.62
10 Old World temperate	73	3.35
11 Temperate Asia	16	0.73
12 Mediterranean, West Asia to Central Asia	6	0.28
13 Central Asia	5	0.23
14 East Asia	178	8.16
15 Endemic to China	76	3.48
8–15 (Total temperate elements)	(623)	(28.56)
Total	2,181	100.00

Temperate distributions in total (Types 8‐15) made up 28.56% of the total genera, of which the north temperate distribution contributed 8.71%. Genera in this distribution included *Betula*,* Carpinus*,* Cornus*,* Pinus*,* Salix*,* Sorbus*. I found that 8.16% of the genera belonged to the East Asian distribution and included *Actinidia*,* Belamcanda*,* Cephalotaxus*,* Gardneria*,* Hovenia*,* Pegia*,* Skimmia*,* Stachyurus*. Further, the East Asia and North America disjuncted distribution made up 3.62% of the total; genera in this distribution were *Castanopsis*,* Illicium, Magnolia*,* Mahonia*,* Nyssa*,* Photinia*, S*chisandra*. I found 76 Chinese endemic genera, contributed 3.48%; examples included *Camptotheca*,* Parakmeria*,* Sargentodoxa*,* Tapiscia*,* Tetrapanax*,* Tutcheria*.

The flora of southern China consisted mostly of tropical genera; however, it has 48 families of temperate distribution, contributing to 21.15% of the total families, and 623 temperate genera, making up 28.56% of the total. While the flora of southern China has some features of marginal tropical distribution, it clearly shows the tropical Asian affinity.

### 4.3 Variation in floristic composition and geographical elements of the tropical flora

The flora of southern China shows conspicuous variations in floristic composition from region to region. Despite this, I found that the floristic similarities at the family and generic levels were more than 90% and 64%, respectively, but at the specific level, there is less than 50% similarity among the compared regional floras from southwestern China to southeastern China (Table 5). More similar dominant families and genera exist between southeastern Xizang (Tibet) and southeastern Yunnan, especially the families Ericaceae and Aralicaceae (Zhu, 2017c).

**Table 4:** Comparison of areal types of genera between the tropical floras across southern China

Areal types of genera	Flora of Motuo, Xizang %^a^	Flora of southern Yunnan %^b^	Flora of southeastern Yunnan %^c^	Flora of southwestern Guangxi %^d^	Flora of Hainan %^e^
1 Cosmopolitan	7.60	4.67	4.57	5.43	5.07
2 Pantropic	16.42	20.47	17.69	20.06	23.07
3 Tropical Asia and tropical America disjuncted	2.85	2.50	2.21	2.96	2.88
4 Old World tropics	6.38	9.91	8.55	9.19	11.46
5 Tropical Asia to tropical Australasia	8.14	10.72	6.04	9.98	13.18
6 Tropical Asia to tropical Africa	4.75	5.72	7.00	4.45	6.24
7 Tropical Asia (Indo‐Malaysia)	16.28	27.72	27.34	23.22	23.77
2–7 (Total tropical elements)	(54.82)	(77.03)	(69.00)	(69.86)	(80.5)
8 North temperate	15.06	5.48	8.18	7.71	4.60
9 East Asia and North America disjuncted	5.16	2.58	3.46	3.95	2.49
10 Old World temperate	3.66	2.34	2.36	2.96	1.56
11 Temperate Asia	0.68	0.40	0.44	0.49	0.31
12 Mediterranean, West Asia to Central Asia	0.54	0.24	0.22	0.30	0.16
13 Central Asia	0.14	0.16	0.07	0.00	0.00
14 East Asia	11.94	5.88	8.84	7.11	3.74
15 Endemic to China	0.41	1.21	3.02	2.17	1.48
8–15 (Total temperate elements)	(37.58)	(18.29)	(26.59)	(24.7)	(14.34)
Total	100	100	100	100	100.00

^a^Data from Yang and Zhou (2015); ^b^data from Zhu and Yan (2012); ^c^data from Zhu and Yan (2009); ^d^data from Qin and Liu (2010), the native plants of tropical southwestern Guangxi were abstracted from this reference; ^e^data from Xing et al. (2012).

In comparing floras, the tropical element is the most consistent regional geographical element. However, I found that the floras of southern and southeastern Yunnan possessed higher portions of tropical Asian elements, whereas the flora of Hainan has the highest ratio of the pantropic element. Additionally, the tropical flora of southeastern Tibet (Xizang) contained the lowest tropical elements of all investigated genera, while the Hainan flora has the highest ratio of the tropical elements (Table 4).

**Table 5:** Comparison of floristic similarities at the family, generic, and specific levels between the tropical floras across southern China

Compared floras	Flora of Motuo, Xizang (159 families, 737 genera, 1,790 species)^b^	Flora of southern Yunnan (192 families, 1,240 genera, 4,150 species)^c^	Flora of southeastern Yunnan (191 families, 1,350 genera, 4,987 species)^d^	Flora of southwestern Guangxi (182 families, 1,006 genera, 2,681 species)^e^	Flora of Hainan (196 families, 1,282 genera, 3,893 species)^f^
Similarity coefficients at family level^a^					
Flora of Motuo, Xizang	100				
Flora of southern Yunnan	93.71	100			
Flora of southeastern Yunnan	99.37	93.19	100		
Flora of southwestern Guangxi	94.97	93.41	95.05	100	
Flora of Hainan	91.19	92.71	89	90.66	100
Similarity coefficients at generic level^a^					
Flora of Motuo, Xizang	100				
Flora of southern Yunnan	72.86	100			
Flora of southeastern Yunnan	83.31	80.81	100		
Flora of southwestern Guangxi	64.99	75.45	84.59	100	
Flora of Hainan	64.31	72.02	67.47	71.47	100
Similarity coefficients at specific level^a^					
Flora of Motuo, Xizang	100				
Flora of southern Yunnan	32.74	100			
Flora of southeastern Yunnan	39.94	53.33	100		
Flora of southwestern Guangxi	18.99	40.1	48.71	100	
Flora of Hainan	17.99	36.4	32.6	34.13	100

^a^Similarity coefficient between A and B = the number of taxa shared by both A and B divided by the lowest number of taxa of A or B, multiplied by 100%.

^b,c,d,e,f^Data from references as Table 4.

### 4.4 Comparison to the floras of Indochina countries

The flora of southern China has distinct similarities at both the family and generic levels with the floras of Indochina countries (Table 6). They have similar coefficients at the family level and share more than 96%, and at generic level more than 80%. The family level coefficients between southern China and across Indochina countries are nearly identical; however, at the generic level, the highest similarity exists between southern China and Laos, up to 85.22%. The tropical flora of southern China undoubtedly belongs to the same biogeographical unit as those of Indochina countries.

**Table 6:** Comparison of floristic similarities at the family and generic levels between the tropical flora of southern China and Indochina countries

Compared flora	Vietnam (231 families, 2,018 genera)	Laos (188 families, 1,373 genera)	Thailand (201 families, 1,475 genera)	Myanmar (220 families, 1,903 genera)
Similarity coefficients at family level (%)^a^				
Tropical flora of China	96.48	97.87	97.01	96.36
Similarity coefficients at generic level (%)^a^				
Tropical flora of China	80.48	85.22	80.75	80.35

a Similarity coefficient between A and B = the number of taxa shared by both A and B divided by the lowest number of taxa of A or B, multiplied by 100%.

## 5 DISCUSSION

The differences seen between the characteristics and evolution of tropical floras of southern China could be influenced in large part by geohistorical events associated with the uplift of the Himalayas, such as the southeastward extrusion of the Indochina geoblock, the clockwise rotation and southeastward movement of Lanping–Simao geoblock, the divergent geological histories between southern and southeastern Yunnan during the Tertiary, as well as the southeastward movement of Hainan Island.

**Figure 2:**
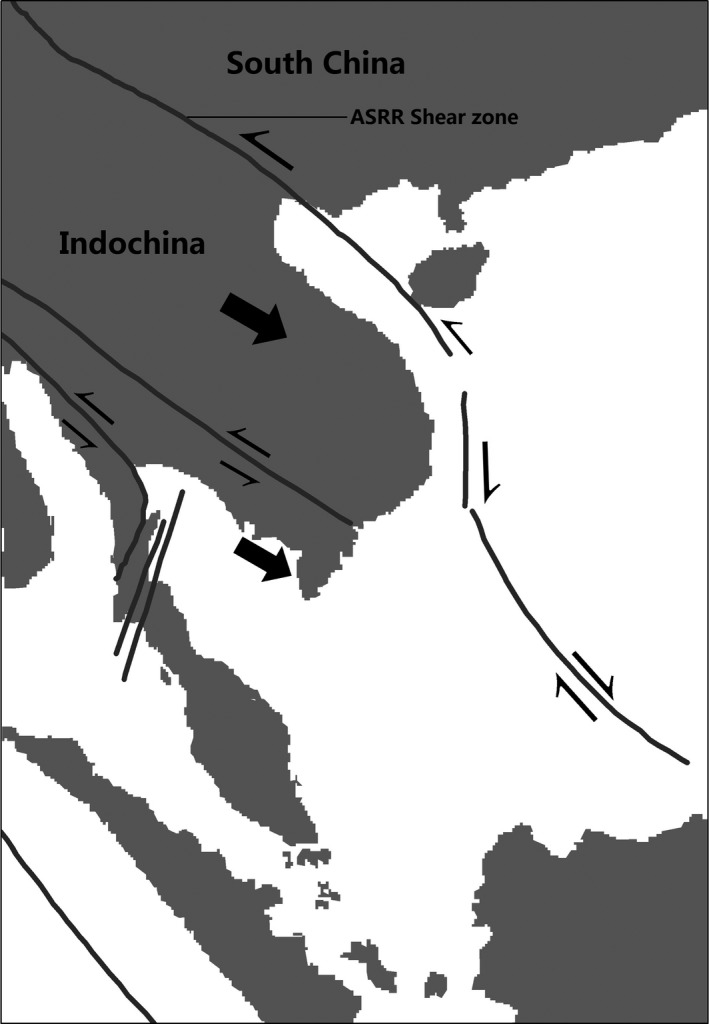
Tectonic model for the Tertiary evolution of strike–slip faults and Indochina excursion in SE Asia. (The figure was redrawn from Morley, 2002; Figure 2, by the Landscape Ecology Lab., Xishuangbanna Tropical Botanical Garden, CAS)

**Figure 3:**
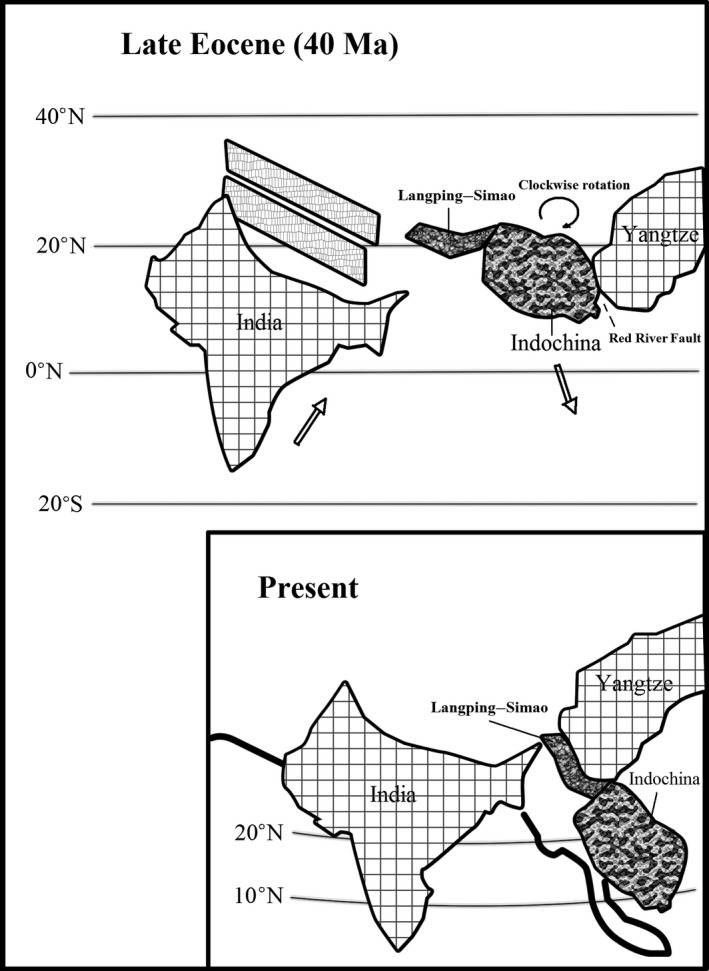
Clockwise rotation and southeastward extrusion of Langping–Simao and Indochina geoblocks during late Eocene (Redrawn from Sato et al., 2001, Figure 7)

The Indian continent collided with Asia around 50 Ma (Rowley, 1996) causing the uplift of the Himalayas, resulting in the continuous deformation of southwestern China, a large clockwise rotation, and southeastward extrusion of Indochina (Chen, Dobson, Heller, & Hao, 1995; Funahara, Nishiwaki, Murata, Otofuji, & Wang, 1993; Harrison, Chen, Leloup, Ryerson, & Tapponnier, 1992; Leloup et al., 1995; Morley, 2002) (Figure 2). The Simao Terrane, which forms the present western and southern parts of Yunnan in southwestern China, has been suggested as one of the prominent fragments of the extruded Indochina block (Sato, Liu, Zhu, Yang, & Otofuji, 1999, 2001; Sato et al., 2007). As a whole, the Simao Terrane was displaced southward by 800 km and rotated clockwise 30° (Figure 3). The rotation processes are believed to have remained active until at least the Miocene (Chen et al., 1995; Schärer et al., 1990). Such geological events may have directly affected the evolution of the flora of southwestern China (Zhu, 2012, 2013b, 2015).

Paleomagnetic studies indicate that during the Mesozoic, Hainan Island was in effect connected to North Vietnam and Guangxi (Mo & Shi, 1987). Blocks reconstruction of Asia reveals that Hainan Island was in a position adjacent to North Vietnam and Guangxi at 40 Ma (Replumaz & Tapponnier, 2003). From the late Mesozoic to early Cenozoic, the Beibu Gulf lithosphere was drawn away, and Hainan moved southeast along the Red River fault and revolved clockwise to its present location (Ma et al., 2014). The Red River fault system also gave rise to the Tonkin Gulf in a widespread extension across a 100‐km‐wide zone prior to 30 Ma (Rangin, Klein, Roques, Le Pichon, & Van Trong, 1995). This evidence suggests the geological evolution of the Tonkin‐Beibu Gulf may have caused Hainan Island's southeast moment. Rock magnetism and paleomagnetism show that Hainan was 5–6° north of its present geographic position in the late Cretaceous (Fu et al., 2010; Liu & Morinaga, 1999). However, it has also been suggested that the rotation of Hainan Island may have occurred during the mid‐Tertiary, when large‐scale left‐lateral motion occurred along the Red River fault as a result of the collision of the Indian Plate into Eurasia, causing extrusion of the Indochina block and the opening of the South China Sea (Li, Metcalfe, & Wang, 1995). Recent biogeographical evidence suggests that Hainan Island may have been in contact with northern Vietnam and Guangxi during the Eocene and drifted to its present location by moving southeast due to the extrusion of the Indochina block (Zhu, 2016a).

In this study, dominant families and genera shared higher similarities between southeastern Tibet (Xizang) and southeastern Yunnan could be explained by the geological history in southwestern China. In the late Eocene India collided with northern Myanmar and Tibet and since the late Cretaceous, northern Myanmar and Tibet have moved northward relative to the Asian plate to the east (Mitchell, 1993). With the southeastward extrusion of the Indochina geoblock, the Lanping–Simao geoblock experienced clockwise rotation and southeastward movement (Chen et al., 1995; Funahara et al., 1993; Harrison et al., 1992; Leloup et al., 1995; Sato et al., 1999, 2001, 2007) causing southeastern Yunnan to move further southward related to the northwestern Yunnan. These events may have shaped the distribution pattern along the so‐called Tanaka line (Tanaka, 1954; Zhu & Yan, 2003) and may have resulted in more similar dominant families and genera, especially between southeastern Xizang (Tibet) (contacting with northwestern Yunnan) and southeastern Yunnan. The floras of southern and southeastern Yunnan have higher portions of the tropical Asian elements compared with other tropical floras in China, which could be the influence of the southeastward extrusion of the Indochina geoblock.

**Figure 4:**
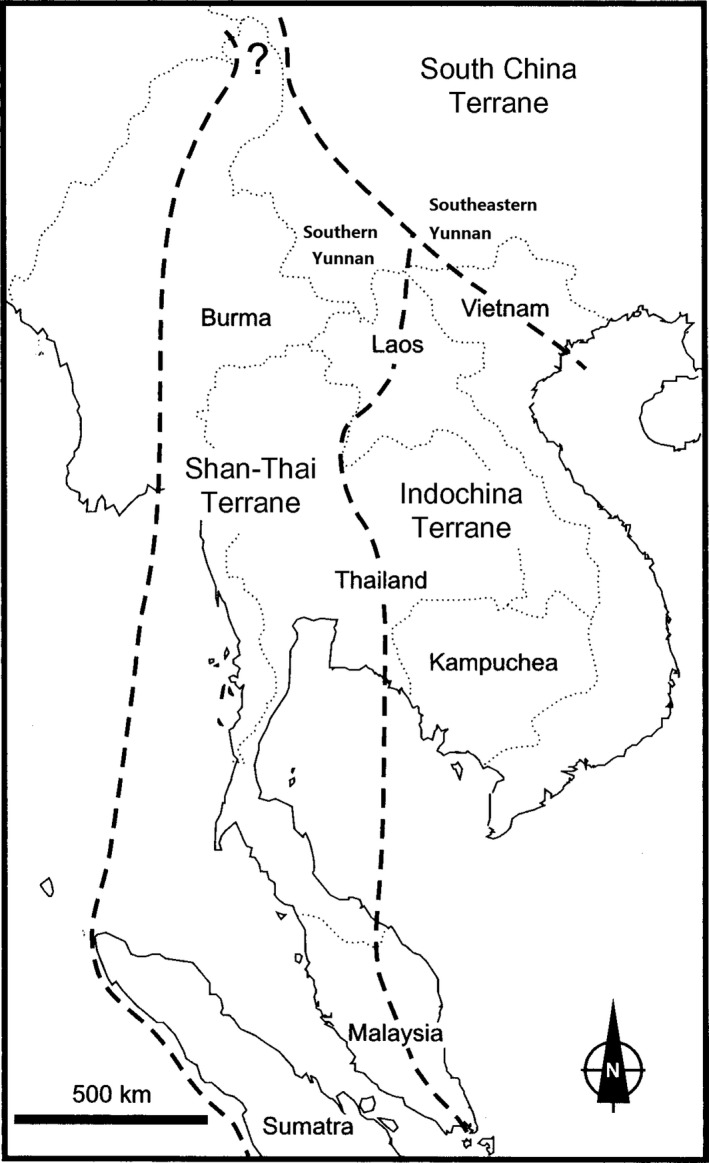
Main geoblocks in Southeast Asia (from Fortey & Cocks, 1998)

Although the two regions share 80% of their genera, 237 genera are restricted to southern Yunnan, and 349 genera to tropical southeastern Yunnan. Furthermore, 57 genera exhibit an East Asian distribution, 53 genera show a north temperate distribution, 22 genera are endemic to China, and 17 genera display an East Asia and North American disjuncted distribution and are found only in tropical southeastern Yunnan (Zhu, 2013b). The flora of southeastern Yunnan is more closely related to eastern Asian flora, while the flora of southern Yunnan is more closely related to Indo‐Malaysian flora. Despite the proximity of the southern and southeastern Yunnan flora, they display distinct differences suggesting they originated from different geoblocks and perhaps a biogeographical boundary should exist between them (Figure 4). This biogeographical line between the tropical floras of southern and southeastern Yunnan was suggested as the “Hua line” (Zhu, 2011, 2013b). Evidence for this biogeographical line is presented by Zhang et al. (2012) using a cluster analysis of species’ presence/absence in Yunnan and their phylogenetic relatedness, taxonomic composition, and regional phylogenetic structure (Li, Kraft, Yang, & Wang, 2015).

After the southeast movement resulting from the uplift of the Himalayas and the extrusion of the Indochina block from its location near Vietnam and Guangxi during the Eocene, the present Hainan Island has a typical tropical climate at 18°10′04″–20°9′40″N in the southernmost of China, which results in the highest number of tropical elements in its flora (Zhu, 2016a). The flora of Taiwan appears to mainly derive from the East Asian flora due to the uplifting of the island after the Late Tertiary, although tropical flora is found in the southernmost part of Taiwan with its tropical climate (Zhu, 2016b).

The uplift of the Himalayas influenced global climate and other environmental changes (Raymo & Ruddimen, 1992; Shi, Li, & Li, 1998; Shi et al., 1999). The uplift of the Himalayas 2.4 Ma occurred quickly with an increase in elevation of 6000 m. This increase in elevation is believed to have created the eastern Asian monsoon climate leading to the tropical and subtropical climate currently occurring on the southwestern China lowland, and thus initiating the evolution of the tropical flora and vegetation in these southern regions of China (Liu, Zhang, & Yuan, 1998). Therefore, the origin and evolution of the tropical flora and vegetation in southern China are related to the geological events and climate changes associated with the uplift of the Himalayas in the southern regions of China.

Due to the clear similarities at the family and generic levels between the tropical flora of southern China and those of the Indochina countries, it appears the tropical flora of southern China has a close affinity with these countries and biogeographically belongs to the same floristic unit in the viewpoint.

## 6 CONCLUSION

The tropical region in China has generally been recognized as the area on the northern edge of tropical Asia, and includes southeastern Xizang (Tibet), southern Yunnan, southwestern Guangxi, southern Guangdong, southern Taiwan, and Hainan Island. Based on present floristic records and data from these tropical areas of China, 12,844 species of seed plants included in 2,181 genera and 227 families are recognized. The families are mainly distributed in tropical areas and extend into temperate zones and contribute to the majority of the flora of southern China. Genera with tropical distributions also make up a greater part of the total flora indicating that the flora of southern China is of marginal tropics. In geographical elements, the genera with tropical Asian distributions comprise the highest proportion among the various distribution types, supporting a tropical Asian or Indo‐Malaysia affinity of the tropical flora of China. Additionally, the tropical flora of China shows conspicuous variations in floristic composition and geographical elements from region to region due to different geological history and ecological environments. Despite this, the floristic similarities at the family and generic levels are more than 90% and 64%, respectively, but lower than 50% at specific level among the compared regional floras from southwestern China to southeastern China. I found that there are more similar dominant families and genera, and also higher similarities at these levels between southeastern Xizang (Tibet) and southeastern Yunnan. The floras of southern and southeastern Yunnan have higher portions of the tropical Asian elements compared with other tropical floras in China, although they have the highest similarity at the specific level. The dominant families and genera have noticeable differences between them; specifically, the flora of Hainan has the highest ratio of tropical elements, of which the pantropic element has the highest portion. Evidently, the differences in characteristics and evolution of these regional tropical floras could very well be influenced by geohistorical events associated with the uplift of the Himalayas, such as the southeastward extrusion of the Indochina geoblock, clockwise rotation, and southeastward movement of Lanping–Simao geoblock, divergent geological histories between southern and southeastern Yunnan, and southeastward movement of Hainan Island. The comparison of the similarity coefficients between the flora of southern China and Indochina countries shows 96% and 80% similarity at the family and generic levels, respectively, indicating their close floristic affinity and inclusion in the same biogeographical floristic unit.

## ACKNOWLEDGMENTS

This project was funded by The National Natural Science Foundation of China (41471051, 31170195, 41071040). The database was made by Yan Lichun from Xishuangbanna Tropical Botanical Garden, Chinese Academy of Sciences. English editing was completed by TopEdit (www.topedit.cn). The author would like to the thank reviewers’ constructive suggestions on this article.

## CONFLICT OF INTEREST

None declared.

## AUTHOR CONTRIBUTIONS

HZ contributed to the conceptualization, data curation, formal analysis, funding acquisition, investigation, methodology, project administration, resources, validation visualization, writing‐original draft, and writing‐review and editing.
